# T-cell epitope vaccine design by immunoinformatics

**DOI:** 10.1098/rsob.120139

**Published:** 2013-01

**Authors:** Atanas Patronov, Irini Doytchinova

**Affiliations:** Department of Chemistry, Faculty of Pharmacy, Medical University of Sofia, Sofia, Bulgaria

**Keywords:** T-cell epitopes, major histocompatibility-binding prediction, immunoinformatics

## Abstract

Vaccination is generally considered to be the most effective method of preventing infectious diseases. All vaccinations work by presenting a foreign antigen to the immune system in order to evoke an immune response. The active agent of a vaccine may be intact but inactivated (‘attenuated’) forms of the causative pathogens (bacteria or viruses), or purified components of the pathogen that have been found to be highly immunogenic. The increased understanding of antigen recognition at molecular level has resulted in the development of rationally designed peptide vaccines. The concept of peptide vaccines is based on identification and chemical synthesis of B-cell and T-cell epitopes which are immunodominant and can induce specific immune responses. The accelerating growth of bioinformatics techniques and applications along with the substantial amount of experimental data has given rise to a new field, called immunoinformatics. Immunoinformatics is a branch of bioinformatics dealing with *in silico* analysis and modelling of immunological data and problems. Different sequence- and structure-based immunoinformatics methods are reviewed in the paper.

## 2. Introduction

The word ‘vaccination’ was used for first time by Edward Jenner in 1796 to describe the injection of smallpox vaccine [1]. Louis Pasteur developed the concept through his innovative work in microbiology. Now, vaccination is the administration of antigenic agents applied to stimulate the immune system of an individual and to develop adaptive immunity to a disease. Vaccines can ameliorate, or often even prevent, the effects of infection. Vaccination is generally considered to be the most effective method of preventing infectious diseases [2], and the efficacy of vaccination has been extensively studied and verified [3–5]. The administration of some vaccines is conducted after the patient has already been infected by the pathogen. Vaccination conducted after exposure to smallpox, within the first 3 days, is reported to attenuate the disease considerably, and administration up to a week after exposure is able to provide some protection from disease, or may ease its severity [6]. Also, a multi-stage tuberculosis vaccine has recently been developed to confer protection after the exposure to the pathogen [7]. There are numerous vaccine examples, including experimental ones against AIDS, cancer and Alzheimer's disease. The core mechanism behind all the vaccinations is the ability of the vaccine to initiate an immune response in a quicker fashion than the pathogen itself.

The purpose of every vaccination is to present a particular antigen or set of antigens to the immune system in order to evoke a relevant immune response. The main active component of a vaccine may be inactive, but still intact (attenuated bacteria or viruses), or purified components of the pathogen that are known to induce immune reaction.

## 3. Types of vaccines

### 3.1. Inactivated vaccines

This type of vaccine consists of virus particles grown in cell culture and inactivated by applying high temperature or chemicals such as formaldehyde. The viral particles are unable to replicate because they are destroyed, but the capsid proteins of the virus have remained intact enough to be recognized and used by the immune system in order to induce a response. If properly produced, the vaccine is not a threat; however, if the inactivation is not performed successfully, active infectious particles can be administered together with the vaccine. Additional booster shots are often needed in order to secure the immune response, because the properly produced vaccine cannot reproduce inside the host.

### 3.2. Live attenuated vaccines

The attenuated vaccines contain live virus particles with low levels of virulence. They have retained their ability to slowly reproduce, and thus they remain a continuous source of antigen for a certain period after the first vaccination, reducing the need of booster shots to keep the antigen levels sufficiently high. Such vaccines are produced by passing virus in cell cultures, in animals or at suboptimal temperatures, allowing selection of less virulent strains or by mutagenesis, or targeted deletions in genes required for virulence [8–10].

### 3.3. Subunit vaccines

Subunit vaccines use only the antigenic components that best stimulate the immune system, instead of dealing with the entire micro-organism. The fact that the subunit vaccine content is mainly represented by the essential antigens reduces the chances of adverse reactions to the vaccine. A subunit vaccine introduces an antigen to the immune system without involving any viral particles. The number of antigens in subunit vaccine can range from 1 to 20 or more. Of course, the identification of the most promising antigens to stimulate the immune system is often a time-consuming process, and can be very difficult. Subunit vaccines are often known for causing weaker antibody responses in comparison with the other vaccine classes. One of the most successful subunit vaccines is the hepatitis B vaccine containing the surface antigen HbsAg [11,12].

### 3.4. Virus-like particles

Virus-like particle (VLP) vaccines are comprised only of viral proteins that take part in the assembly of the virus structure. They have the ability to self-assemble into virus resembling the particles from which they were derived without the presence of the viral nucleic acid, which makes them simply non-pathogenic [13,14]. By contrast with the subunit vaccines, VLPs usually have higher immunogenicity owing to their multi-valent and highly repetitive structure. VLPs have been produced from a broad range of viruses that belong to Retroviridae, Flaviviridae and Parvoviridae families. Vaccines against viruses such as human papillomavirus and hepatitis B are VLP-based vaccines that are currently in clinical use [15]. Additionally, a pre-clinical vaccine against chikungunya virus was developed based on the same approach [16]. VLPs are typically produced in a variety of cell cultures, such as mammalian cell lines, insect cell lines, and plant and yeast cells [17].

### 3.5. Toxoid vaccines

The toxoid vaccines are typical solution for bacteria that secrete harmful metabolites or toxins. It is common to use them when the main reason for discomfort or sickness is a bacterial toxin. Such toxoid vaccines are produced by treating the toxins with formalin, thus inactivating them, and still retaining their structure for further recognition by the immune system. Examples of toxoid vaccines are the vaccines against diphtheria and tetanus.

### 3.6. DNA vaccines

DNA vaccination is a very new approach for induction of humoral and cellular immune responses to protein antigens by administering genetically engineered DNA. The majority of DNA vaccines are still in the experimental stage, and have been tested in numerous viral, bacterial and parasitic models of disease, and also in a few tumour models. DNA vaccines represent an innovative approach for immunization, bringing a number of advantages over conventional vaccines and giving the possibility of inducing a broader variety of immune response types [18–25]. The risks of DNA vaccines are limited [22]. Several groups demonstrated that cancer vaccines can be effective for the induction of specific immunity against cancer-associated antigens without negative side effects like integration of plasmid DNA into the host genomes or induction of pathogenic anti-DNA antibodies [23–25].

### 3.7. Peptide vaccines

The improved knowledge of antigen recognition at molecular level has contributed to the development of rationally designed peptide vaccines. The general idea behind the peptide vaccines is based on the chemical approach to synthesize the identified B-cell and T-cell epitopes that are immunodominant and can induce specific immune responses. B-cell epitope of a target molecule can be conjugated with a T-cell epitope to make it immunogenic. The first epitope-based vaccine was created in 1985 by Jackob *et al*. [26]. They introduced recombinant DNA and express epitopes against cholera in *Escherichia coli*. Epitope-based vaccines can be constructed for T and B lymphocytes [27,28]. The T-cell epitopes are typically peptide fragments, whereas the B-cell epitopes can be proteins, lipids, nucleic acids or carbohydrates [27–31]. Peptides have become desirable vaccine candidates owing to their comparatively easy production and construction, chemical stability, and absence of infectious potential. The peptide vaccines against various cancers have been developed, and entered phase I and phase II of clinical trials, with satisfactory clinical outcome. The peptide vaccination is commonly being studied for application in both ameliorating and prophylactic immunotherapy [32]. Yet there is more to be improved in order to eliminate obstacles, such as the need for a better adjuvant and carrier or the low immunogenicity. Nonetheless, current efforts are showing much promise in defying these limitations and providing improvements for this approach.

## 4. T-cell epitopes

**Figure 1.**
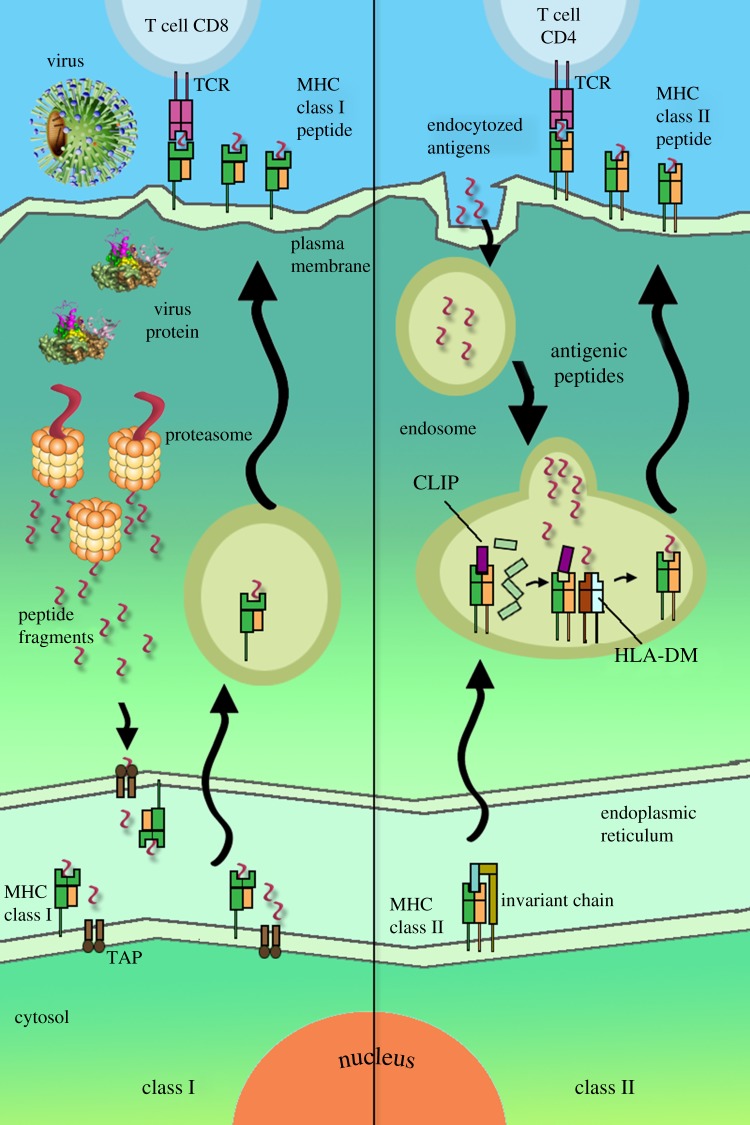
Antigen-processing pathways in the cell. Left: intracellular pathway. Protein is cleaved into oligopeptides in the proteasome, the peptides enter the endoplasmic reticulum (ER) via TAP protein and bind to MHC class I, and the complex peptide–MHC protein is presented on the cell surface. Right: extracellular pathway. Protein is endocytozed, cleaved into oligopeptides in the endosome, bound to MHC class II protein and presented on the cell surface. In the ER, MHC class II molecules are adjoined to a specific peptide, known as invariant chain (Ii). It blocks the binding cleft of the MHC molecule, thereby preventing the binding of endogenous peptides. In the endosome, the Ii is initially cleaved to CLIP peptide, and is then replaced by an exogenous peptide. The process is facilitated by the HLA-DM molecule.

The epitope is recognizable by the immune system part of the antigen, and in particular by antibodies, B cells or T cells. The epitopes may belong to both foreign and self proteins, and they can be categorized as conformational or linear, depending on their structure and integration with the paratope [33]. T-cell epitopes are presented on the surface of an antigen-presenting cell (APC), where they are bound to major histocompatibility (MHC) molecules in order to induce immune response [34]. MHC class I molecules usually present peptides between 8 and 11 amino acids in length, whereas the peptides binding to MHC class II may have length from 12 to 25 amino acids [35]. MHC class II proteins bind oligopeptide fragments derived through the proteolysis of pathogen antigens, and present them at the cell surface for recognition by CD4^+^ T cells (figure 1). If sufficient quantities of the epitope are presented, the T cell may trigger an adaptive immune response specific for the pathogen. Class II MHCs are expressed on specialized cell types, including professional APCs such as B cells, macrophages and dendritic cells, whereas class I MHCs are found on every nucleated cell of the body [36].

The recognition of epitopes by T cells and the induction of immune response have a key role for the individual's immune system. Even the slightest deviation from the normal functioning can have a grave impact on the organism. In case of autoimmune disease, the T cells recognize the cells' native peptides as foreign, and attack and eventually destroy the organism's own tissues.

Some viruses, such as human immunodeficiency virus (HIV), hepatitis C, and avian and swine influenza, manage to avoid recognition by the T cell relying on various mutations that effectively alter the amino acid sequences of the proteins encoded by the viral genes [37,38].

Knowledge about the peptide's epitopes has a key role for manufacturing epitope-based vaccines, which, injected into the recipient, can induce immune response. One of the key issues in T-cell epitope prediction is the prediction of MHC binding, as it is considered a prerequisite for T cell recognition. All T-cell epitopes are good MHC binders, but not all good MHC binders are T-cell epitopes.

MHCs are among the most polymorphic proteins in higher vertebrates, with more than 6000 class I and class II MHC molecules listed in IMGT/HLA [39]. Determining the peptide-binding preferences exhibited by this extensive set of alleles is beyond the present capacity of experimental techniques, necessitating the development of bioinformatics prediction methodologies. The most successful prediction methods for T-cell epitopes developed to date have been data-driven. T-cell epitope prediction typically involves defining the peptide-binding specificity of specific class I or class II MHC alleles and then predicting epitopes *in silico*. Using peptide sequence data, experimentally determined affinity data have been used in the construction of many T-cell epitope prediction algorithms. Such methods include motif-based systems, support vector machines (SVMs) [40,41], hidden Markov models (HMMs) [42–44], quantitative structure–activity relationship (QSAR) analysis [45,46], and structure-based approaches [47].

## 5. Immunoinformatics

The accelerating growth of bioinformatics techniques and applications along with the substantial amount of experimental data has made a significant impact on the immunology research. This has led to a rapid growth in the field of computation immunology, and a number of immunology-focused resources and software, which help in understanding the properties of the whole immune system, have become available [48]. This has given rise to a new field, called immunoinformatics. Immunoinformatics can be described as a branch of bioinformatics concerned with *in silico* analysis and modelling of immunological data and problems.

Immunoinformatics research stresses mostly on the design and study of algorithms for mapping potential B- and T-cell epitopes, which speeds up the time and lowers the cost needed for laboratory analysis of pathogen gene products. Using such tools and information, an immunologist can analyse the sequence areas with potential binding sites, which in turn leads to the development of new vaccines. The methodology of analysing the pathogen genome to identify potential antigenic proteins is known as ‘reverse vaccinology’ [49]. This is mainly beneficial because conventional methods need to dedicate time to pathogen cultivation and subsequent protein extraction. Although pathogens grow quickly, extraction of their proteins and then testing of those proteins on a large scale is expensive and time-consuming. Immunoinformatics is capable of reducing time and saving resources for the development of relevant vaccines by revealing virulence genes and surface-associated proteins.

Normally, the investigation of the binding affinity of antigenic peptides to the MHC molecules is the main goal when predicting epitopes. The experimental techniques are found to be difficult and time-consuming, and therefore several *in silico* methodologies are being created and constantly improved to identify epitopes. The list of approaches includes matrix-driven methods, QSAR analysis, identification of structural binding motifs, protein threading, homology modelling, docking techniques, and design of several machine-learning algorithms and tools. In the past, computational techniques could only identify sequence characteristics, but new improved algorithms and tools are being designed to increase the predictive performance [49]. The methods used for development of prediction models can be divided into structure-based methods that derive information from the three-dimensional structure of the proteins, and sequence-based methods that analyse the amino acid sequence.

### 5.1. Sequence-based methods

#### 5.1.1. Motif search-based approach

The combination of preferred amino acids at some of the peptide anchor binding positions is called a motif. The motif search is the most outdated, yet the most widely used method for prediction of epitopes [50–53]. The peptide amino acid sequence is searched for motifs by using a motif library [54]. The MHC-binding motifs for a given peptide can be identified by comparison of known binders and non-binders [55]. The motif search approach was used to identify epitopes that bind HLA-DR allele among the proteins expressed by *Plasmodium falciparum* [56]. EPIPREDICT is another motif-based tool, used for the identification of MHC class II-binding epitopes from proteins involved in the human gluten intolerance [57]. D'Amaro *et al*. [58] developed the computer program MOTIF, which yields collection of all the known affinity motifs to HLA-A*0201. The program identifies 27 binders when validated against an external test set, and the subsequent experiments confirm that 18 of these peptides exhibit binding affinity with overall accuracy of 61 per cent. Another tool is EpiMer, created at Brown University and used for prediction of HIV-related epitopes [59,60].

One of the widely used epitope prediction tools is SYFPEITHI, which is also based on the motif search approach [54,61]. Similar to the EpiMer approach, SYFPEITHI is used to score the peptides and evaluate their immunogenicity. Numerous experimental *in vivo* and *in vitro* assays have been conducted to validate the *in silico* predictions [62–70].

The accuracy of the motif-based algorithms is about 60–70 per cent, mostly because not all of the binding peptides have recognizable motifs [71]. In many cases, the correlation between the predicted and the experimentally determined affinities is very weak. A study conducted by Andersen *et al*. [72] compares the affinities predicted by SYFPEITHI and BIMAS binders with the experimentally determined ones from a set of oncogenes and viral proteins. The authors show a large number of wrongly identified false positives, while some of the actual epitopes are predicted as non-binders.

#### 5.1.2. Prediction by artificial neural network

The artificial neural networks (ANNs) provide a convenient method for finding relationships and describing nonlinear data [73]. ANN methods are frequently used by the bioinformatics researchers for solving asthma-related problems [74], to investigate cardiac diseases [75] and drug solubility [76], and for epitope prediction and analysis of MHC haplotypes [77]. When applying for epitope prediction, the peptide length can be highly variable. The sequences included in the training set are usually aligned by assigning a specific anchor position. This is a trivial task when constructing models for MHC I prediction, where the difference in the peptide length is negligible, while it becomes a challenging quest for MHC II, where the length variability is considerably larger.

Nielsen *et al*. [78] described an improved neural network model to predict T-cell class I epitopes. The NETCTL server [79] (http://www.cbs.dtu.dk/services/NetCTL/) uses a method to integrate the prediction of peptide MHC class I binding, proteasomal C-terminal cleavage and transporter associated with antigen processing (TAP) transport efficiency. It has updated from version 1.0 to 1.2 to improve the accuracy of MHC class I peptide-binding affinity and proteasomal cleavage prediction. NETMHC server v. 3.2 [80] (http://www.cbs.dtu.dk/services/NetMHC) is based on ANN and weight matrices. It has been trained on data from 55 MHC peptides (43 human and 12 non-human) and position-specific scoring matrices for a further 67 HLA alleles. MHC class I molecule motifs are well defined, but the prediction of MHC class II binding peptides is considered harder to achieve, mainly because of the variable length of reported binding peptides, the undetermined core region for each peptide and the number of primary anchor amino acids.

#### 5.1.3. Prediction by support vector machine

The SVM is a computer science concept for a set of supervised learning methods used for data analysis and pattern recognition, developed by Vapnik [81] and commonly used for image and data classification and regression analysis [82]. SVMs belong to the group of the kernel-based approaches [83]. Classically, the SVM takes a set of data and predicts, for each given input, to what type of input class it belongs; therefore, SVM is described as a non-probabilistic binary linear classifier. The SVM model can be represented as two sets of points in space, distributed in a way that the two subsets falling into separate categories are divided by a clear gap that is as wide as possible. The model categorizes the novel data points depending on which side of the gap they fall on.

Another formal description of the SVM method is that it defines a hyperplane or set of hyperplanes in a high- or infinite-dimensional space, which can be used for classification, regression or other purposes. The optimal separation can be achieved by deriving the hyperplane that is positioned at the largest distance from the nearest points belonging to any of the modelled classes. The larger the distance, the more reliable is the model [84].

Nanni [85] demonstrated the use of SVM and SV data description to predict T-cell epitopes. In the case of TAPPRED, Bhasin & Raghava [86], analysed nine features of amino acids to find the correlation between binding affinity and physico-chemical properties. They developed an SVM-based method to predict the TAP binding affinity of peptides, and found cascade SVM to be more reliable. Cascade SVM has two layers of SVMs, and its performance is better than the other available algorithms. It is experimentally determined that the immunoproteasome plays a role in the generation of the MHC class I ligand. Often the computational approach is preferred over experimental analysis for studying and predicting the cleavage specificities of proteasomes. Therefore, a web application called PCLEAVAGE [87] has been developed to predict cleavage sites in antigenic proteins. It uses SVM [88], parallel exemplar-based learning [89] and Waikato Environment for Knowledge Analysis [90].

Sweredoski & Baldi [91] presented COBEPRO, which is a two-step system for the prediction of continuous B-cell epitopes. In the first step, COBEPRO assigns a fragment epitopic propensity score to protein sequence fragments using an SVM. In the second step, it calculates an epitopic propensity score for each residue based on the SVM scores of the peptide fragment in the antigenic sequence. It is incorporated into the SCARTCH prediction suite. However, COBEPRO is not able to find the difference between antigen and non-antigen, and in order to increase the efficacy it should be used with high-throughput technologies.

#### 5.1.4. Hidden Markov models

HMMs were initially described in the second half of the 1960s by Baum *et al*. [92]. HMMs were first applied for speech recognition in the mid-1970s [93,94]. In the second half of the 1980s, HMMs found their application in the analysis of biological sequences [95], and in particular of DNA sequences. Since then, they have become ubiquitous in the field of bioinformatics [96].

HMM-based approaches are widely used in bioinformatics and proteomics for the prediction of protein sequences with helical secondary structure [97], transmembrane regions [98,99] and protein homology analysis [100]. HMM is also used for sequence alignment [101], and protein family identification by Pfam and SMART [102]. For the purposes of genomics, HMM is used for studying gene splicing [103], phylogenetic tree analysis [104] and gene identification in procariotes [105].

Zhang *et al*. [106] developed PREDTAP for the prediction of peptide binding to hTAP. They used a three-layer back propagation network with the sigmoid activation function. The inputs were the binary strings, representing nonamer peptide. In addition, they used second-order HMM. The results were both sensitive and specific. Mamitsuka [44] derived HMM-based, high-accuracy models for prediction of peptide-binding affinity to HLA-A*0201 and DR1 proteins. By using Mamitsuka's approach, Udaka *et al*. [107] derived models for other MHC class I proteins. Brusic *et al*. [108] also used HMM for binding affinity prediction towards the HLA-A2 family members. The analysis included only the amino acids involved in a direct interaction with the protein. HMM was derived for each allele of the family, and peptides also binding to the other alleles were used as a training set. The test sets comprised peptides binding to the corresponding allele.

Schonbach *et al*. [109] compare the predictions done by HMM, ANN and quantitative matrices (QMs). Over 500 amino acid sequences of HIV-1 and -2 are scanned for peptides with affinity to A*0201 and B*3501. The ANN model showed high performance for the A*0201 allele, and the HMM was more successful in predicting B*3501 binders. Subsequent experiments showed that 26 per cent of the epitopes were successfully identified by the models based on QMs and ANN.

#### 5.1.5. Prediction by quantitative matrices-driven methods

QMs resemble an extended motif with assigned coefficients for each amino acid at each position in the peptide [110]. In principle, matrix-based epitope prediction can be divided into four steps: first, all possible peptide frames are extracted from a given protein sequence. Second, the corresponding position- and amino acid-specific matrix values are assigned to each residue of a given peptide frame. Next, the side chain values of each peptide are added or multiplied, resulting in the peptide ‘score’. Last, peptides are selected based on their peptide score. Thus, instead of simply counting anchor residues, matrix-based algorithms take into account the relative importance of every amino acid residue in a peptide sequence, as charged by their effect on binding. QMs provide a linear model with easy-to-implement capabilities. Another advantage of using this approach is that it covers a wider range of peptides with binding potential and it gives a quantitative score to each peptide. Their predictive accuracies are also considerable. The capacity to predict HLA class II ligands using QM-based algorithms was first demonstrated for DRB1*0401 molecules [111,112]. These algorithms ranked naturally processed peptides and T-cell epitopes in the top 2–4 per cent of all possible peptide frames of given antigens, even if they owned only one or two anchor residues. More important, however, a correlation between the peptide score and the binding affinity was demonstrated [111], which therefore supports the underlying approximation that a given residue contributes to binding independently of its neighbouring amino acid residues. Later on, many more QM-based algorithms were established, including algorithms for DRB1*0101, DRB1*1501, DRB1*1101, DRB1*0701 and DRB1*0801 molecules. The predictive power of some of these algorithms was validated by a computer simulating the screening of M13 peptide display libraries. QM-based algorithms were used instead of purified HLA-class II molecules to enrich for large class II-binding peptide repertoires [113].

QMs are also applied for the prediction of cleavage sites and are implemented in MAPPP [114]. Similar algorithms are applied for the prediction of linear epitopes of the B lymphocytes. Alix [115] calculates the molecular properties for the 20 common amino acids (side chain flexibility, hydrophilic affinity and accessible surface) and uses these properties for the prediction of potential epitope regions in the proteins that would possibly bind to the B cells.

BIMAS is a T-cell epitope prediction server that implements algorithms based on QM [116]. BIMAS was used for the identification of various potential epitopes [64,70,117,118]. QM was derived from experimental data from the dissociation half-time of the MHC–peptide complexes. The model predicting binding to HLA-A*0201 allele is based on the author's data, and the models for the other alleles are based on the literature data. Servers such as BIMAS and SYFPEITHI are shown to perform well in the prediction of known epitopes, but are accurate enough when screening proteins in search for unknown and novel epitopes [69].

Another QM-based model is EpiMatrix, developed at Brown University [59]. It has been used for the identification of HIV-1 antigens [59,119]. Other similar approaches are implemented in ClustiMer and Conservatrix. ClustiMer identifies promiscuous (for a given HLA superfamily) peptides, and Conservatrix determines unchanged (conserved) regions in the proteins of the mutant pathogens of the same species [120].

Another category of QMs is the position-specific matrices, where the frequency at which the given amino acid appears at a certain position is calculated for binding and non-binding to MHC peptides [121]. Nielsen *et al*. [78] derive QM for MHC class I and II epitopes accounting for the changes in the Gibbs energy.

Virtual matrix (VM) is another type of QM, created by Sturniolo *et al*. [122]. VM models the interactions between each amino acid and the pockets of the binding groove. The advantage comes from the applicability of the VM to different alleles that share similar structural characteristics of the binding groove, whereas the QMs are strictly specific to the given allele. TEPITOPE is VM-based and predicts peptides that are HLA-DR binders. TEPITOPE is used for identification of epitopes in the tumour antigen MAGE-3 [123,124]. Another tool using VM is ProPred, created by Singh & Ragava [125], where the profiles of the MHC protein pockets created by Sturniolo served as a foundation for the models.

MHCPred is a sequence-based server using the additive method [126] for developing QMs. The additive method derives QMs using multiple linear regression by partial least-squares (PLS) method. MHCPred was used to design superbinders [127] and to identify the first T-cell epitope binding to HLA-Cw*0102, and originating from HIV proteome [128].

EpiJen is a multi-step algorithm for T-cell epitope prediction. It models the four steps of antigen processing—cleavage in the proteasome, binding to TAP protein, binding to MHC protein and recognition by T cells [129]. For each step, a QM was developed and arranged in a consecutive mode to select only those peptides that will be generated by the proteasome, transported by TAP, bound in MHC and recognized by T cells. In the final set are collected the peptides most probably acting as T-cell epitopes.

VaxiJen predicts immunogenicity of whole proteins. It includes five models derived by PLS-based discriminant analysis, which covers the bacterial, viral, tumour, parasite and fungal kingdoms [130]. The models show accuracy between 70 and 97 per cent.

EpiTOP is a server for MHC class II-binding prediction based on proteochemometrics [131]. Proteochemometrics is a QSAR method specially designed to deal with ligands binding to a set of similar proteins [132]. The structures of the target proteins are described by proper descriptors and enter the X matrix of QSAR. The affinity of a peptide to a particular MHC protein is considered as a function of the structures of both binding peptide and target protein. EpiTOP is among the top three best-working servers for MHC class II-binding prediction [131].

The main drawback of the quantitative models is that they are strongly dependent on the type, number and quality of the data that comprise the training set of peptides. The inclusion of novel data often alters the values upon which the QM is based. Brusic *et al*. [133] suggest as a prerequisite a threshold value for the derivation of a reliable model to be 150 peptides and the ideal size of training set should reach 600 peptides. However, in reality, most of the alleles are represented by scarce data rarely exceeding more than 50 peptides. This limits the range of applicability for this approach to the alleles that are sufficiently well studied.

### 5.2. Structure-based methods

The structure-based methods do not solely rely on binding data and sequence information, but rather use the structural information, and use computational methods developed in the field of structural biology for prediction of potentially good binders.

For the MHC molecule to recognize antigenic peptides, geometric and electrostatic complementarities between the receptor and ligand are essential for the formation of a stable complex. Many computational studies that attempt to unravel the rules governing peptide binding to MHC use the sequences of MHC-binding peptides. By aligning the sequences known to bind to a given MHC molecule, residues favouring the binding could be identified along the peptide. The synthesis of this knowledge together with that obtained from crystallographic studies has led to better understanding of the basic principles that guide peptide–MHC recognition [134,135].

#### 5.2.1. Docking of peptides and screening of peptide libraries

Over recent years, many techniques and methods, such as combinatorial peptide library screening and ligand docking, commonly used in the drug design field, have found their application for the purposes of bioinformatics. Davenport *et al*. [136] generated MHC class II models by evaluating the contribution of a given amino acid to the overall peptide affinity. They took into account how frequently the amino acid is present at a certain position. New peptides exhibiting affinity towards DRB1*0101 were found based on relationships derived from peptide libraries [137]. Screening of peptide libraries was also applied for studying other MHC alleles. Stryhn *et al*. [138] analysed the peptide specificities of MHC class I binders by using peptide libraries. Stevens *et al*. [139] used peptide libraries to determine the preferred peptide length for murine MHC alleles. By using the positional screening of combinatorial peptide libraries, Udaka *et al*. [140,141] characterize the peptides binding to H-Kb Db and Ld alleles. The different amino acids were screened for how frequently they appear at the different positions of the peptides from the training set, and QMs were generated in order to predict the affinity of the peptides from the test set. The accuracy of the predictions reached 80 per cent. Similar studies were conducted by Sung *et al*. [142] and Nino-Vasquez *et al*. [143].

Computer-simulated ligand docking is a quick and powerful technique for investigating intermolecular interactions. In general, the purpose of docking simulation is twofold: to find the most probable translational, rotational and conformational juxtaposition of a given ligand–receptor pair and to evaluate the relative binding affinity of the ligand towards its receptor.

Docking is mostly known for its wide application in computer-aided drug design [144]. However, this approach found its application for designing novel peptides exhibiting binding affinity towards MHC. Initially, the docking studies were mainly used for investigation of peptides that bind MHC class I molecules [145,146]. Zeng *et al*. [147] used residues with different properties (polar, hydrophobic, charged, etc.) by docking them to different positions of the binding groove of the receptor, thus evaluating the most acceptable residues' properties for each position of the potential epitope. Another study [148] uses a genetic algorithm in order to derive QM for A2 and A24 alleles, and peptides with high binding affinity are designed. The peptide structures were modelled and docked to the binding groove. The binding energy was calculated as a sum of the electrostatic and hydrophobic components. After the experimental determination of the peptides' binding affinity, good correlation is observed between the predicted and the experimentally derived values.

Docking is also used for studying peptides binding MHC class II alleles for identification of anchor positions and positions that are solvent-exposed [149]. The interaction between the T-cell receptor and the MHC–ligand complex were also studied via docking [150,151]. Tong *et al*. [152] develop a novel docking approach that consists of three steps: (i) anchor residue docking; (ii) positioning of the peptide backbone in the binding groove; and (iii) adjustment of the overall positioning of the peptide backbone and the side chains. This approach showed improved accuracy in comparison with the other methods. Liu *et al*. [67] take into account the flexibility of the MHC proteins during the docking simulation. However, despite the high predictive accuracy, these methods are not feasible for online predictions since the time required for the simulation is unreasonably long. Furthermore, the accuracy of the predictions is highly dependent on the quality of the structural information available for the receptor and the correctly modelled backbone of the ligand.

EpiDOCK is a structure-based server for MHC-binding prediction of peptides using docking score-based QMs (DS-QMs) [153]. It predicts binding to 12 HLA-DR, 6 HLA-DQ and 5 HLA-DP proteins.

#### 5.2.2. Application of threading algorithms

Knowledge-based threading algorithms are used to discriminate the binding and non-binding peptides for particular MHC molecules without relying on previous data. The algorithm usually takes into account the contributions of individual amino acids along the peptide that prompt them to fit into the binding groove of MHC molecule using knowledge-based contact potential [154]. Often, the accurate prediction of peptide structure in the MHC-binding groove is hindered owing to the limited availability of suitable peptide backbone templates. Still, the applicability of the threading algorithm can be extended to a larger number of MHC alleles for the prediction of T-cell epitope by using molecular modelling methods on the peptide–MHC complex. Although the treading is not capable of exact modelling of peptide in the MHC groove, it can verify the probability of a peptide sequence to adopt a particular fold in the MHC groove using binding energy score [155–157].

Adrian *et al*. [155] studied the MHC complex–peptide interactions, and reveal the significant role played by the peptide's backbone for the overall binder's selection. They also stress the significance of exact knowledge about the ligand's conformation and its impact on the ability to produce more accurate prediction models. They use threading to predict the peptides’ conformations by remodelling them over the existing backbone known from an X-ray study of MHC complexes. The scores used to evaluate the overall binding affinity are additively calculated by summing the individual binding energy score of each amino acid residue at each position [158]. The lower values correspond to higher affinity [156,157].

The drawback of this method is that despite the high level of overlapping between the referent and the tested peptides, some residue side chains tend to be oriented in different directions, and thus worsen the predictability. Additional modelling, however, may improve the predictive accuracy of the model [157].

#### 5.2.3. Binding energy and molecular dynamics

The epitopes can be identified by calculating the change in the free Gibbs energy during the formation of the complex between the ligand and the receptor, which is defined as the difference between the energy of the free and the bound peptide [159,160]. The epitopes can be found by direct comparison of the free energies of two peptides by using scoring functions or molecular dynamics (MD) simulations [161]. MD is used for studying the binding of synthetic peptides [162], MHC peptide–protein complexes [163,164], the role of the water molecules involved in the formation of the peptide–protein complex [165], the interactions between A2 peptides and the receptor's binding groove [161,166], the dissociation of the MHC–peptide complexes [167], and the interactions between the T-cell receptor and the peptide–MHC protein complex [168]. Rognan *et al*. [163] simulated the binding of six peptides to B*2705 protein and showed the importance of the secondary anchor residues. Lim *et al*. [169] simulated the interaction between the peptide and HLA-A*0201 protein by using the available X-ray structure. The peptides predicted to have high binding affinity were validated experimentally. In another study, MD is used to identify the contribution of each residue at a given position and the results are used to form a QM for epitope prediction [147]. Analogous MD simulations are performed in order to determine anchor residues for the HLA-A*0217 allele [170]. MD simulations are used for studying peptides binding to DRB1 [171]. Davies *et al*. [172] built epitope prediction models for MHC class II proteins by using simulated annealing, a common optimization method where the peptide conformation is obtained by rapid increase of the temperature and subsequent recalculation of the protein coordinates by gradually decreasing the temperature at each step. The energy of the resulting complex is derived and used for binding affinity predictions.

Another approach is to derive the binding energy as a difference between the energy of the solvated complex and the energies of the solvated binding partners—peptide and protein receptor. Only the electrostatic and hydrophobic terms are taken into account [173].

Different scoring functions can be used for the evaluation of the interactions between the peptide and the MHC protein. The advantage of this approach is that it delivers more accurate information about which types of interactions govern the stability of the complex [174,175]. Sezerman *et al*. [159] generate free energy maps describing the binding sites along the binding groove of the MHC class I proteins by using the electrostatic energy, solvation energy and the conformational entropy terms of the amino acid side chains. Froloff *et al*. [176] calculate the binding energy for eight peptide MHC class I protein complexes based on polar and non-polar interactions. Schapira *et al*. [173] calculate the binding energy based on three terms—entropic, electrostatic and hydrophobic potentials—and use it for predicting the formation of small protein complexes.

The free energy calculation approach was also applied on peptides binding to HLA-A*0201 [177]. They used an energy evaluation function where the free-binding energy consists of five terms: hydrogen bond energy between the peptide and the receptor, interaction energy between the hydrophobic atoms, entropic loss upon binding, decrease of the binding energy upon interaction between polar and non-polar atoms, and the transition energy required for the transport of an atom between environments with different dielectric constants. For another experiment, Rognan and co-workers [174] used the Fresno method for prediction of the free-binding energy. The training set includes five known binders interacting with HLA-A*0201; there is X-ray data and complex affinity data available for the complexes. Based on the free complex energy, a model is derived to predict the affinity of 26 more binders to the HLA-A*0204 allele that shares significant structure similarities with HLA-A*0201. The study shows, however, that the predictive accuracy is much higher when there is structural information available about the receptor. This approach was used for estimation of the binding energy of peptides binding to A*0201 and B*2705 by using the available X-ray structures [174]. Later on, the Fresno approach is applied to build the peptide MHC–protein complexes via homology modelling and to calculate the binding energy [175]. The main drawback of this method is the amount of time and computational power that it takes to produce results, which makes it inapplicable for online access.

## 6. Conclusion

Immunoinformatics can effectively leverage computational techniques to deliver effective and utilitarian advantage in the search of new vaccines. It is considered to contribute to vaccine design as the computational chemistry contributes to drug design. Immunoinformatics-based vaccine design is able to achieve effective, cost-efficient development of vaccines or vaccine components.

## 7. Acknowledgements

The authors thank their colleagues from the Faculty of Pharmacy, the Medical University of Sofia—Ivan Dimitrov, Mariyana Atanasova and Panaiot Garnev—for their contributions in the developing of EpiTOP, AllerTOP and EpiDOCK. I.D. thanks her former colleagues from the Jenner Institute, Oxford University—Darren R. Flower, Pigping Guan, Channa Hattotuwagama and Martin Blythe—for their contributions in the developing of MHCPred, EpiJen and VaxiJen. Part of this work was supported by the National Research Fund of the Bulgarian Ministry of Education and Science (grant no. 02-1/2009).
